# Gastric cancer: immunohistochemical classification of molecular subtypes and their association with clinicopathological characteristics

**DOI:** 10.1007/s00428-017-2240-x

**Published:** 2017-10-19

**Authors:** Eva-Maria Birkman, Naziha Mansuri, Samu Kurki, Annika Ålgars, Minnamaija Lintunen, Raija Ristamäki, Jari Sundström, Olli Carpén

**Affiliations:** 1 Department of Pathology, University of Turku, Kiinamyllynkatu 10, 20520 Turku, Finland; 2 Department of Pathology, Turku University Hospital, Kiinamyllynkatu 10, 20520 Turku, Finland; 3 Auria Biobank, University of Turku and Turku University Hospital, Kiinamyllynkatu 8, 20520 Turku, Finland; 4 Department of Oncology, Turku University Hospital, Hämeentie 11, 20520 Turku, Finland; 5 MediCity Research Laboratory, University of Turku, Tykistökatu 6, 20520 Turku, Finland; 6 Pathology, Research Programs Unit and HUSLAB, University of Helsinki and Helsinki University Hospital, Haartmaninkatu 3, 00014 Helsinki, Finland

**Keywords:** Gastric cancer, Immunohistochemistry, In situ hybridisation, Molecular classification

## Abstract

Gastric cancer is traditionally divided into intestinal and diffuse histological subtypes, but recent molecular analyses have led to novel classification proposals based on genomic alterations. While the intestinal- and diffuse-type tumours are distinguishable from each other at the molecular level, intestinal-type tumours have more diverse molecular profile. The technology required for comprehensive molecular analysis is expensive and not applicable for routine clinical diagnostics. In this study, we have used immunohistochemistry and in situ hybridisation in molecular classification of gastric adenocarcinomas with an emphasis on the intestinal subtype. A tissue microarray consisting of 244 gastric adenocarcinomas was constructed, and the tumours were divided into four subgroups based on the presence of Epstein-Barr virus, TP53 aberrations and microsatellite instability. The intestinal- and diffuse-type tumours were separately examined. The distribution of *EGFR* and *HER2* gene amplifications was studied in the intestinal-type tumours. Epstein-Barr virus positive intestinal-type tumours were more common in male patients (*p* = 0.035) and most often found in the gastric corpus (*p* = 0.011). The majority of the intestinal-type tumours with TP53 aberrations were proximally located (*p* = 0.010). All tumours with microsatellite instability showed intestinal-type histology (*p* = 0.017) and were associated with increased overall survival both in the univariate (*p* = 0.040) and multivariate analysis (*p* = 0.015). In conclusion, this study shows that gastric adenocarcinomas can be classified into biologically and clinically different subgroups by using a simple method also applicable for clinical diagnostics.

## Introduction

Gastric cancer is one of the major causes of cancer-related death worldwide [1]. Gastric adenocarcinomas have traditionally been divided into intestinal and diffuse subtypes according to the Laurén classification based on the histological characteristics of the tumours [2]. However, The Cancer Genome Atlas Consortium (TCGA) has recently proposed a molecular subtyping of gastric adenocarcinomas based on the presence of Epstein-Barr virus (EBV), microsatellite instability (MSI), genomic stability (GS) and chromosomal instability (CIN) [3]. The majority of the GS tumours have diffuse histology, while the other subgroups contain predominantly intestinal-type tumours. An alternative classification has been proposed by the Asian Cancer Research Group (ACRG). This proposal stratifies gastric adenocarcinomas into tumours with MSI, microsatellite-stable tumours showing epithelial to mesenchymal transition (MSS/EMT), MSS tumours with intact TP53 activity (MSS/TP53+) and MSS tumours with functional loss of TP53 (MSS/TP53−) [4]. These classification systems have provided valuable information about the variability in biological characteristics among gastric adenocarcinomas. Instead of considering gastric cancer as a single disease, it has also become clear that when exploring new cancer therapies, future studies need to be conducted among defined sets of patients whose tumours have specific genomic abnormalities. In clinical practice, HER2 is the only predictive biomarker for targeted therapy currently used for patient selection in gastric cancer.

The complex methodologies used in these abovementioned studies are not applicable for routine clinical diagnostics, and some more straightforward methods have already been proposed [5–8]. In this study, we have constructed a next-generation tissue microarray (ngTMA) from 244 intestinal- and diffuse-type adenocarcinomas of the stomach, gastro-oesophageal junction (GOJ) and distal oesophagus [9]. Using this cohort, we have been able to identify four subgroups of tumours with distinct molecular and clinicopathological characteristics by combining the Laurén classification, MSI and TP53 immunohistochemistry (IHC) and EBER in situ hybridisation (ISH). Additionally, the analysis of E-cadherin expression was performed in order to compare our method with the previously published studies, and the distribution of *EGFR* and *HER2* gene amplifications were examined among these subgroups in the intestinal-type tumours.

## Materials and methods

### Patients and tumour specimens

The study population consists of 244 patients diagnosed with adenocarcinoma of the stomach, GOJ or distal oesophagus at the Turku University Hospital between years 1993 and 2012. The initial search in the clinical database of Auria Biobank retrieved tumour specimens from 437 patients. The exclusion criteria were carcinoma in situ (Tis, *n* = 23), insufficient sample material or indeterminate histology (*n* = 63), neuroendocrine histology (*n* = 3) and metastatic adenocarcinoma from a different organ (*n* = 6). This resulted in 190 patients with intestinal-type tumours and 152 patients with diffuse-type tumours. From these, all intestinal-type tumours and 54 representative diffuse-type tumours were included in the ngTMA. Among all of the patients, 11.9% (29/244) did receive preoperative chemotherapy (13 patients with intestinal-type and 16 with diffuse-type tumours). The type of surgery was total gastrectomy for 154 (63.1%) patients, subtotal gastrectomy or tumour resection for 72 (29.5%) patients and palliative surgery for 18 (7.4%) patients. The extent of surgery was determined as R0 (no residual tumour) for 180 (73.8%) patients, R1 (microscopic residual tumour) for 34 (13.9%) patients and R2 (macroscopic residual tumour) for 20 (8.2%) patients. The extent of surgery could not be determined for 10 (4.1%) patients. Tumour stage was assessed according to the current WHO Classification manual [10]. The median follow-up time of the patients was 125 months. All corresponding H&E slides have been reviewed for confirmation of diagnosis and adequacy of material. Relevant clinical information has been gathered from each case. The reporting of the study has been performed in compliance with the current recommendations [11]. The patient characteristics are presented in Table 1.

**Table 1:** Patient characteristics

Number of patients	All, *n* (%)	Intestinal, *n* (%)	Diffuse, *n* (%)
All	244	190 (77.9)	54 (22.1)
Median age at diagnosis (range)			
	72.3 (32.9–90.9)	74.4 (32.9–90.9)	66.8 (36.9–85.1)
Patient sex			
Female	101 (41.4)	68 (35.8)	33 (61.1)
Male	143 (58.6)	122 (64.2)	21 (38.9)
Site of primary tumour^a^			
Distal oesophagus	19 (7.8)	19 (10.0)	
GOJ/cardia	60 (24.6)	60 (31.6)	
Corpus	106 (43.4)	52 (27.4)	
Antrum/pylorus	59 (24.2)	59 (31.1)	
Tumour differentiation grade			
Grade 1	17 (7.0)	17 (8.9)	0 (0)
Grade 2	93 (38.1)	93 (48.9)	0 (0)
Grade 3	134 (54.9)	80 (42.1)	54 (100.0)
Stage			
I	46 (18.9)	40 (21.1)	6 (11.1)
II	102 (41.8)	79 (41.6)	23 (42.6)
III	83 (34.0)	61 (32.1)	22 (40.7)
IV	13 (5.3)	10 (5.3)	3 (5.6)
Follow-up status			
Alive and free of disease	48 (19.7)	34 (17.9)	14 (25.9)
Alive with disease	1 (0.4)	1 (0.5)	0 (0)
Deceased	195 (79.9)	155 (81.6)	40 (74.1)

*GOJ* gastro-oesophageal junction

^a^Diffuse-type tumours are included in the tumours of the corpus

### Tissue microarray construction

The ngTMA was created as follows [9]. The appropriate formalin-fixed paraffin-embedded (FFPE) tissue specimens were chosen based on clinical data and retrieved from the pathology archives. A representative haematoxylin-eosin (H&E) section containing areas of invasive carcinoma was selected from each tumour. New H&E slides were produced, scanned (Pannoramic P250, 3DHistech) and uploaded into the university digital microscopy web portal (casecenter.utu.fi). Each slide was viewed using Pannoramic Viewer software (3DHistech). Using the 1.0-mm annotation tool, annotations of different colours corresponding to various histological areas were placed onto each digital slide. Two annotations were placed in the centre of the tumour and two annotations in the periphery or invasive front of the tumour. The corresponding tissue cores were then transferred into the TMA blocks using an automated TMA instrument (TMA Grandmaster, 3DHistech) by overlaying each annotated digital slide with the corresponding tissue specimen. One tissue core containing benign tissue was selected from each tumour to act as a control. The constructed TMA blocks were sectioned, stained, scanned and uploaded into the web portal (casecenter.utu.fi), and each individual spot was scored by two pathologists (EB and NM). The resulting scores were combined with the clinical data for statistical analysis.

The link https://seafile.utu.fi/d/7c4aa1964b/ contains examples of our TMA results. The directory “TMA staining” contains low resolution images of all stainings performed on TMA block number 7, and “TMA map and score” contains the map and scores of the same TMA block. The directory “Example scoring” contains selected high resolution images of positive/negative and aberrant/wild-type stained tissue cores.

### Immunohistochemistry and in situ hybridisation

**Fig. 1:**
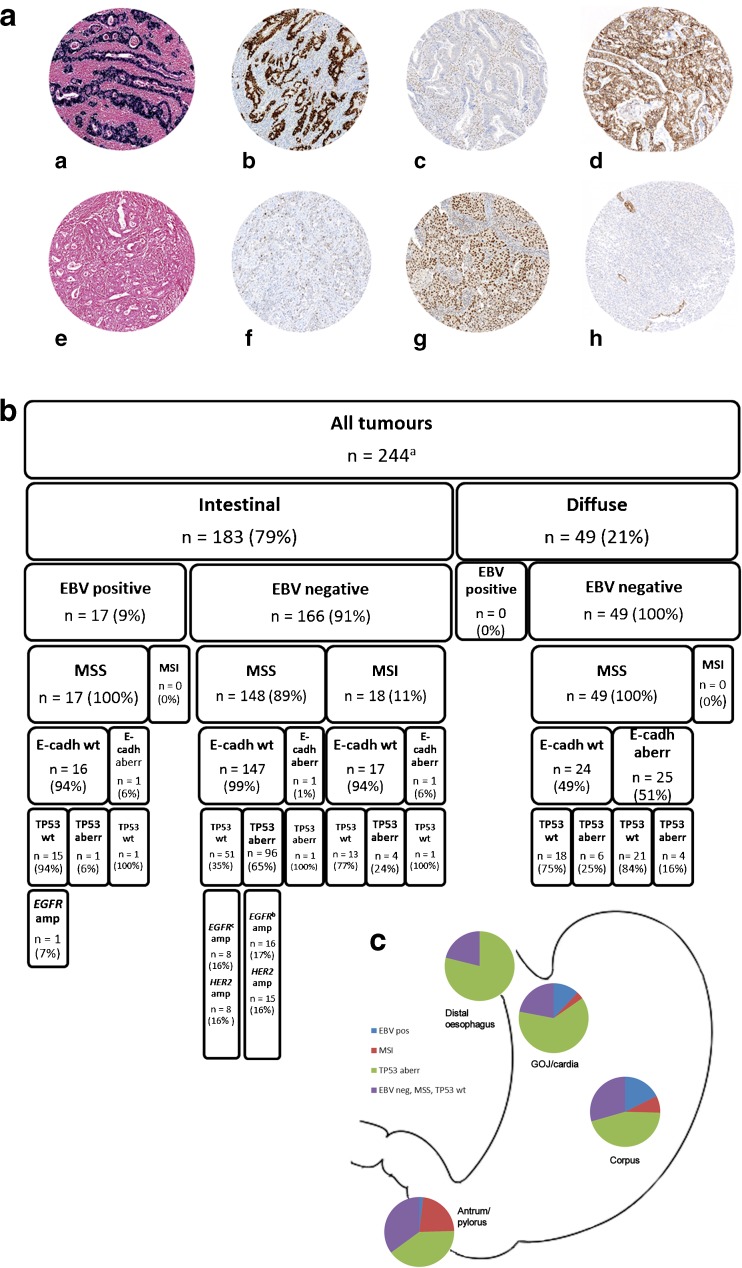
Classification of adenocarcinomas of the stomach, gastro-oesophageal junction and distal oesophagus based on immunohistochemistry and in situ hybridisation. **a** Examples of EBER in situ hybridisation and MLH1, TP53 and E-cadherin immunohistochemistry in eight oesophagogastric adenocarcinomas. (a) EBV positive, (b) TP53 aberration, (c) MLH1 mutated, (d) E-cadherin wild-type, (e) EBV negative, (f) TP53 wild-type, (g) MLH1 wild-type and (h) E-cadherin aberration. **b** The classification of the intestinal- and diffuse-type adenocarcinomas of the stomach, gastro-oesophageal junction and distal oesophagus according to the immunohistochemical data and in situ hybridisation. **c** Distribution of the four molecular subtypes of intestinal-type oesophagogastric adenocarcinomas in different anatomical locations. ^a^Immunohistochemical data available for 238 (EBV, MSI, TP53) or 232 tumours (E-cadherin). ^b^Including one tumour (2%) with co-amplification *EGFR* and *HER2*. ^c^Including five tumours (5%) with co-amplification of *EGFR* and *HER2*. EBV Epstein-Barr virus, GOJ gastro-oesophageal junction, MSS microsatellite-stable, MSI microsatellite-instable, wt wild-type, aberr aberration, amp amplification

IHC reactions were performed on 4-μm paraffin sections of each tumour on the TMA slides with BenchMark XT (Ventana/Roche). For TP53, a ready-to-use antibody clone Bp53-11 (Ventana/Roche) was used, and the protocol included mild (30 min) CC1 pretreatment together with 28-min antibody incubation. Signal detection was performed with ultraView universal DAB Detection Kit (Ventana/Roche). For MLH1, an antibody clone G168-15 (BD Pharmingen) was used at 1:5 dilution together with standard (60 min) CC1 pretreatment and 36-min antibody incubation. The signal was detected with ultraView Universal DAB Detection Kit and amplification kit. For MSH2, an antibody clone G219-1129 (BD Pharmingen) was used at 1:200 dilution together with standard CC1 pretreatment and 28-min antibody incubation. The signal was detected with ultraView Universal DAB Detection Kit. For MSH6, an antibody clone EP49 (Epitomoc) was used at 1:200 dilution together with standard CC1 pretreatment and 32-min antibody incubation. The signal was detected with ultraView Universal DAB Detection Kit. For PMS2, a ready-to-use antibody clone EPR3947 (Ventana/Roche) was used together with extended (90 min) CC1 pretreatment, 44-min antibody incubation and 12-min HQ LINKER and HRP MULTIMER enhancements. The signal was detected with OptiView Universal DAB Detection Kit and amplification kit. For EBV, a ready-to-use EBER (EBV-encoded small RNA) probe (Ventana/Roche) was used together with ISH-Protease 3 pretreatment for 28 min and 1-h probe incubation. The signal was detected with ISH iVIEW Blue Detection Kit. For E-cadherin, an antibody clone NCH-38 (Agilent Technologies) was used at 1:100 dilution together with standard CC1 pretreatment and 32-min antibody incubation. The signal was detected with ultraView Universal DAB Detection Kit and amplification kit. A complete loss of or strong diffuse TP53 nuclear positivity was classified as aberrant TP53 expression. EBER ISH was scored either positive or negative according to the nuclear reaction. A tumour was classified as MSI if at least one of the markers (MLH1, MSH2, MSH6 and PMS2) showed a complete loss of nuclear reactivity together with positive background reaction in benign epithelium, smooth muscle cells and lymphocytes. Negative nuclear reactivity with negative background was considered controversial and not used for classification Loss of membranous reactivity or only faint cytoplasmic reaction was classified as aberrant E-cadherin expression (Fig. 1a). The methods for EGFR and HER2 IHC and silver in situ hybridisation (SISH) have been described previously [12–15]. In short, with EGFR the scoring was based on the most intense membranous or membranous+cytoplasmic staining (0, negative; 1+, weak; 2+, moderate; 3+, strong). Specimens were classified as IHC high if showing 2+ or 3+ membranous or membranous+cytoplasmic staining intensity in ≥ 10% of tumour cells. With HER2 IHC, specimens showing 2+ or 3+ membranous staining in ≥ 10% of tumour cells were classified as IHC high. The IHC high samples were further analysed with SISH. The EGFR and HER2 IHC and SISH were performed on whole tissue sections.

### Statistical analysis

Statistical analyses were performed with IBM SPSS Statistics for Windows, version 21.0 (IBM Corporation, Armonk, NY). Frequency table data were analysed using Pearson’s *χ* ^2^ test or Fisher’s exact test for categorical variables and 2 × 2 tables were used to calculate odds ratios (OR). The Kaplan-Meier method and log-rank test as well as Cox’s proportional hazards regression model were used for univariate survival analysis. Multivariate survival analysis was performed by Cox’s proportional hazards regression model. Recurrence-free survival (RFS) was calculated from the time of diagnosis to the time of first recurrence, death of any cause or to the last follow-up date. Only recurrences ≥ 6 months after the time of diagnosis were considered relevant. Detection of a local or distant recurrence < 6 months from the diagnosis was considered likely to present an initially advanced disease. Patients without disease recurrence ≥ 6 months after diagnosis were considered curatively treated. Overall survival (OS) was calculated from the time of diagnosis to the time of death of any cause or the last follow-up date. Five patients (2.0%) who had received trastuzumab treatment for recurrent cancer were excluded from the OS analysis and additionally 13 patients with stage IV disease (5.3%) from the RFS analysis. All statistical tests were two-sided, and *p* values under 0.05 were considered statistically significant.

## Results

### MSI, TP53 and E-cadherin immunohistochemistry and EBV in situ hybridisation

EBV, MSI and TP53 were analysed in 238 tumours and E-cadherin in 232 tumours. In the other tumours, the markers could not be evaluated due to insufficient tissue material. EBV RNA was found to be present in 17/186 (9.1%) of the intestinal tumours, while none of the diffuse tumours was EBV positive (Fisher’s exact test, *p* = 0.028; RR 0.91, 95% CI 0.87–0.95). MSI was detected in 19/186 (10.2%) of the intestinal tumours, while none of the diffuse tumours was found to have MSI phenotype (Fisher’s exact test, *p* = 0.017; RR 0.90, 95% CI 0.86–0.94). Aberrant TP53 expression was observed to be significantly more common among intestinal-type (103/186, 55.4%) than diffuse-type tumours (10/52, 19.2%) (Fisher’s exact test, *p* < 0.0001; OR 5.21, 95% CI 2.47–11.0). Ninety-four tumours (39.5%) were found to be EBV negative, MSS and TP53 wild-type. Of these, 52/186 (28.0%) tumours had intestinal-type and 42/52 (80.8%) tumours had diffuse-type histology (Fisher’s exact test, *p* < 0.0001; OR 0.09, 95% CI 0.04–0.20). None of the MSI tumours had both EBV positivity and aberrant TP53 expression. Among the intestinal-type tumours, 3/183 (1.6%) tumours had aberrant E-cadherin expression, whereas among the diffuse-type tumours, aberrant E-cadherin expression could be seen in 25/49 (51.0%) tumours. Among the EBV negative, MSS and TP53 wild-type tumours, 21/39 (53.8%) of the diffuse-type tumours but none of the intestinal-type tumours (*n* = 51) had aberrant E-cadherin expression.

Among the intestinal-type tumours, aberrant TP53 expression was more common in EBV negative than in EBV positive tumours (Fisher’s exact test, *p* < 0.0001; OR 0.041, 95% CI 0.01–0.32). Tumours with aberrant TP53 expression were also more frequently MSS than MSI (Fisher’s exact test, *p* = 0.003; OR 5.46, 95% CI 1.74–17.2). No association was found between aberrant E-cadherin expression and either EBV, TP53 or MSI status. Among the diffuse-type tumours, testing for statistical significance was not applicable for EBV or MSI (Table 2, Fig. 1b).

**Table 2:** Results from immunohistochemical stainings and EBER in situ hybridisation

		TP53, *n* (%)		*p* value^a^	EBV, *n* (%)		*p* value^a^	MMR, *n* (%)		*p* value^a^	E-cadherin, *n* (%)		*p* value^a^	Combined, *n* (%)		*p* value^a^
		aberr	wt		pos	neg		MSI	MSS		aberr	wt		EBV neg, TP53 wt, MSS	Others	
Intestinal		103 (55.4)	83 (44.6)		17 (9.1)	169 (90.9)		19 (10.2)	167 (89.8)		3 (1.6)	180 (98.4)		52 (28.0)	134 (72.0)	
TP53, *n* (%)	aberr				1 (5.9)	102 (60.4)	*< 0.0001*	4 (21.1)	99 (59.3)	*0.003*	1 (33.3)	101 (56.1)	0.585			
	wt				16 (94.1)	67 (39.6)		15 (78.9)	68 (40.7)		2 (66.7)	79 (43.9)				
EBV, *n* (%)	pos	1 (1.0)	16 (19.3)	*< 0.0001*				0 (0)	17 (10.2)	0.225	1 (33.3)	16 (8.9)	0.255			
	neg	102 (99.0)	67 (80.7)					19 (100.0)	150 (89.8)		2 (66.7)	164 (91.1)				
MMR, *n* (%)	MSI	4 (3.9)	15 (18.1)	*0.003*	0 (0)	19 (11.2)	0.225				1 (33.3)	17 (9.4)	0.268			
	MSS	99 (96.1)	68 (81.9)		17 (100.0)	150 (88.8)					2 (66.7)	163 (90.6)				
E-cadherin, *n* (%)	aberr	1 (1.0)	2 (2.5)	0.585	1 (5.9)	2 (1.2)	0.255	1 (5.6)	2 (1.2)	0.268				0 (0)	3 (2.3)	0.561
	wt	101 (99.0)	79 (97.5)		16 (94.1)	164 (98.8)		17 (94.4)	163 (98.8)					51 (100.0)	129 (97.7)	
Diffuse		10 (19.2)	42 (80.8)		0 (0)	52 (100.0)		0 (0)	52 (100.0)		25 (51.0)	24 (49.0)		42 (80.8)	10 (19.2)	
TP53, *n* (%)	aberr				0 (0)	10 (19.2)	NA	0 (0)	10 (19.2)	NA	4 (16.0)	6 (25.0)	0.496			
	wt				0 (0)	42 (80.8)		0 (0)	42 (80.8)		21 (84.0)	18 (75.0)				
EBV, *n* (%)	pos	0 (0)	0 (0)	NA				0 (0)	0 (0)	NA	0 (0)	0 (0)	NA			
	neg	10 (100.0)	42 (100.0)					0 (0)	52 (100.0)		25 (100.0)	24 (100.0)				
MSI, *n* (%)	MSI	0 (0)	0 (0)	NA	0 (0)	0 (0)	NA				0 (0)	0 (0)	NA			
	MSS	10 (100.0)	42 (100.0)		0 (0)	52 (100.0)					25 (100.0)	24 (100.0)				
E-cadherin, *n* (%)	aberr	4 (40.0)	21 (53.8)	0.496	0 (0)	25 (51.0)	NA	0 (0)	25 (51.0)	NA				21 (53.8)	4 (40.0)	0.496
	wt	6 (60.0)	18 (46.2)		0 (0)	24 (49.0)		0 (0)	24 (49.0)					18 (46.2)	6 (60.0)	
All tumours		113 (47.5)	125 (52.5)		17 (7.1)	221 (92.9)		19 (8.0)	219 (92.0)		28 (12.1)	204 (87.9)		94 (39.5)	144 (60.5)	

*n* = 238 for TP53, EBV and MMR and *n* = 232 for E-cadherin

*aberr*, aberrant; *EBV*, Epstein-Barr virus; *MMR*, mismatch repair; *MSI*, microsatellite-instable; *MSS*, microsatellite-stable; *NA*, not applicable; *neg*, negative; *pos*, positive; *wt*, wild-type

^a^Fisher’s exact test. *p* values < 0.05 are considered statistically significant and shown in italics

### EGFR and HER2 in the intestinal-type adenocarcinomas

EGFR and HER2 protein expression levels were evaluated in 183 intestinal-type adenocarcinomas. Moderate/strong EGFR protein expression was found in 59 (32.2%) of the tumours and 25 (13.7%) of the tumours had moderate/strong HER2 protein expression. Among these, *EGFR* gene amplification was detected in 27/59 (14.8% of the whole study material) tumours and *HER2* gene amplification in 24/25 (13.1% of the whole study material) tumours. No significant associations were observed between EGFR/HER2 protein expression level or gene amplification and TP53/EBV/MSI status. The majority of the *EGFR* (*n* = 17) or *HER2* (*n* = 15) gene amplifications were found in the group of EBV negative and MSS tumours with aberrant TP53. Most of the co-amplifications (*n* = 5) were also found in this subgroup (Fig. 1b). The co-localisation of aberrant TP53 expression and either *EGFR* or *HER2* gene amplification was detected more often in the proximal (distal oesophagus/GOJ/cardia) than distal (corpus/antrum/pylorus) intestinal-type tumours (Fisher’s exact test, *p* = 0.019; OR 2.83, 95% CI 1.21–6.61).

### TP53, EBV and MSI status in relation to clinicopathological variables

Among the intestinal-type tumours, aberrant TP53 expression was more frequent in proximal than distal tumours (Fisher’s exact test, *p* = 0.002; OR 2.71, 95% CI 1.47–5.00). In contrast, MSI phenotype was more frequent in distally located tumours (Fisher’s exact test, *p* = 0.003; OR 7.10, 95% CI 1.59–31.7). EBV positive tumours were more common among male than female patients (Fisher’s exact test, *p* = 0.035; OR 4.57, 95% CI 1.01–20.6), whereas MSI tumours were more common among female than male patients (Fisher’s exact test, *p* = 0.042; OR 2.89, 95% CI 1.07–7.36). EBV positive tumours were also more often poorly differentiated than well or moderately differentiated (Fisher’s exact test, *p* < 0.0001; OR 0.08, 95% CI 0.02–0.37) and most often located in the gastric corpus (Fisher’s exact test, *p* = 0.011). No significant associations were observed between the EBV negative/MSS/TP53 wild-type tumours and the examined clinicopathological variables. Among diffuse-type tumours, no significant associations were found with the examined variables (Table 3).

**Table 3:** EBV, MSI and TP53 status in relation to clinicopathological variables (*n* = 238)

	EBV, *n* (%)		*p* value^a^	MMR, *n* (%)		*p* value^a^	TP53, *n* (%)		*p* value^a^	Combined, *n* (%)		*p* value^a^
	pos	neg		MSI	MSS		aberr	wt		EBV neg, TP53 wt, MSS	Others	
Intestinal												
Patient sex												
Female	2 (11.8)	64 (37.9)	*0.035*	11 (57.9)	55 (32.9)	*0.042*	34 (33.0)	32 (38.6)	0.445	21 (40.4)	45 (33.6)	0.398
Male	15 (88.2)	105 (62.1)		8 (42.1)	112 (67.1)		69 (67.0)	51 (61.4)		31 (59.6)	89 (66.4)	
Histological grade												
Grade 1	0 (0)	17 (10.1)	*< 0.0001*	1 (5.3)	16 (9.6)	0.596	10 (9.7)	7 (8.4)	0.365	6 (11.5)	11 (8.2)	0.378
Grade 2	2 (11.8)	88 (52.1)		8 (42.1)	82 (49.1)		54 (52.4)	36 (43.4)		28 (53.8)	62 (46.3)	
Grade 3	15 (88.2)	64 (37.9)		10 (52.6)	69 (41.3)		39 (37.9)	40 (48.2)		18 (34.6)	61 (45.5)	
Tumour location												
Distal oesophagus/GOJ/cardia	7 (41.2)	71 (42.0)	> 0.999	2 (10.5)	76 (45.5)	*0.003*	54 (52.4)	24 (28.9)	*0.002*	17 (32.7)	61 (45.5)	0.137
Corpus/antrum/pylorus	10 (58.8)	98 (58.0)		17 (89.5)	91 (54.5)		49 (47.6)	59 (71.1)		35 (67.3)	73 (54.5)	
Tumour location												
Distal oesophagus	0 (0)	19 (11.2)	*0.011*	0 (0)	19 (11.4)	*0.002*	15 (14.6)	4 (4.8)	*0.010*	4 (7.7)	15 (11.2)	0.396
GOJ/cardia	7 (41.2)	52 (30.8)		2 (10.5)	57 (34.1)		39 (37.9)	20 (24.1)		13 (25.0)	46 (34.3)	
Corpus	9 (52.9)	42 (24.9)		4 (21.1)	47 (28.1)		23 (22.3)	28 (33.7)		15 (28.8)	36 (26.9)	
Antrum/pylorus	1 (5.9)	56 (33.1)		13 (68.4)	44 (26.3)		26 (25.2)	31 (37.3)		20 (38.5)	37 (27.6)	
T												
T1–T2	5 (29.4)	41 (24.3)	0.768	3 (15.8)	43 (25.7)	0.414	28 (27.2)	18 (21.7)	0.388	13 (25.0)	33 (24.6)	0.958
T3–T4	12 (70.6)	128 (75.7)		16 (84.2)	124 (74.3)		75 (72.8)	65 (78.3)		39 (75.0)	101 (75.4)	
Stage												
I–II	11 (64.7)	104 (61.5)	1.000	13 (68.4)	102 (61.1)	0.624	65 (63.1)	50 (60.2)	0.762	29 (55.8)	86 (64.2)	0.316
III–IV	6 (35.3)	65 (38.5)		6 (31.6)	65 (38.9)		38 (36.9)	33 (39.8)		23 (44.2)	48 (35.8)	
Recurrence												
> 6 months	2 (12.5)	52 (35.4)	0.092	3 (15.8)	51 (35.4)	0.120	32 (35.6)	22 (30.1)	0.506	18 (41.9)	36 (30.0)	0.187
No recurrence	14 (87.5)	95 (64.6)		16 (84.2)	93 (64.6)		58 (64.4)	51 (69.9)		25 (58.1)	84 (70.0)	
EGFR IHC^b, c^												
0–1	14 (82.4)	108 (66.7)	0.275	13 (68.4)	109 (68.1)	> 0.999	62 (62.6)	60 (75.0)	0.106	36 (73.5)	86 (66.2)	0.375
2–3	3 (17.6)	54 (33.3)		6 (31.6)	51 (31.9)		37 (37.4)	20 (25.0)		13 (26.5)	44 (33.8)	
*EGFR* gene amplification^c^												
Yes	1 (5.9)	25 (15.4)	0.474	0 (0)	26 (16.3)	0.080	17 (17.2)	9 (11.3)	0.293	8 (16.3)	18 (13.8)	0.643
No	16 (94.1)	137 (84.6)		19 (100.0)	134 (83.8)		82 (82.8)	71 (88.8)		41 (83.7)	112 (86.2)	
HER2 IHC^b, c^												
0–1	17 (100.0)	138 (85.2)	0.133	19 (100.0)	136 (85.0)	0.081	82 (82.8)	73 (91.3)	0.124	42 (85.7)	113 (86.9)	0.810
2–3	0 (0)	24 (14.8)		0 (0)	24 (15.0)		17 (17.2)	7 (8.8)		7 (14.3)	17 (13.1)	
*HER2* gene amplification^c^												
Yes	0 (0)	23 (14.2)	0.134	0 (0)	23 (14.4)	0.138	15 (15.2)	8 (10.0)	0.372	8 (16.3)	15 (11.5)	0.453
No	17 (100.0)	139 (85.8)		19 (100.0)	137 (85.6)		84 (84.8)	72 (90.0)		41 (83.7)	115 (88.5)	
Diffuse												
Patient sex												
Female	0 (0)	31 (59.6)	NA	0 (0)	31 (59.6)	NA	5 (50.0)	26 (61.9)	0.500	26 (61.9)	5 (50.0)	0.500
Male	0 (0)	21 (40.4)		0 (0)	21 (40.4)		5 (50.0)	16 (38.1)		16 (38.1)	5 (50.0)	
T												
T1–T2	0 (0)	8 (15.4)	NA	0 (0)	8 (15.4)	NA	1 (10.0)	7 (16.7)	0.599	7 (16.7)	1 (10.0)	0.599
T3–T4	0 (0)	44 (84.6)		0 (0)	44 (84.6)		9 (90.0)	35 (83.3)		35 (83.3)	9 (90.0)	
Stage												
I–II	0 (0)	29 (55.8)	NA	0 (0)	29 (55.8)	NA	5 (50.0)	24 (57.1)	0.734	24 (57.1)	5 (50.0)	0.734
III–IV	0 (0)	23 (44.2)		0 (0)	23 (44.2)		5 (50.0)	18 (42.9)		18 (42.9)	5 (50.0)	
Recurrence												
> 6 months	0 (0)	19 (48.7)	NA	0 (0)	19 (48.7)	NA	1 (14.3)	18 (56.3)	0.091	18 (56.3)	1 (14.3)	0.091
No recurrence	0 (0)	20 (51.3)		0 (0)	20 (51.3)		6 (85.7)	14 (43.8)		14 (43.8)	6 (85.7)	

*aberr*, aberrant; *EBV*, Epstein-Barr virus; *GOJ*, gastro-oesophageal junction; *IHC*, immunohistochemistry; *MMR*, mismatch repair; *MSI*, microsatellite-instable; *MSS*, microsatellite-stable; *NA*, not applicable; *neg*, negative; *po*s, positive; *wt*, wild-type

^a^Fisher’s exact test for dichotomous variables and Pearson’s *χ* ^2^ test for other variables. *p* values < 0.05 are considered statistically significant and shown in italics

^b^Strongest membranous staining intensity

^c^ *n* = 179

### TP53, EBV and MSI status in relation to survival

In univariate survival analysis for intestinal tumours, the presence of MSI was associated with longer overall survival (OS, median) (124.6 versus 28.7 months, log-rank test, *p* = 0.040; Cox test, *p* = 0.043, HR 0.54, 95% CI 0.30–0.98) but not with recurrence-free survival (RFS). In intestinal tumours, increasing depth of tumour invasion was associated with shorter RFS and OS (RFS log-rank test, *p* = 0.045; Cox test, *p* = 0.046, HR 1.53, 95% CI 1.01–2.32; OS log-rank test, *p* = 0.030; Cox test, *p* = 0.031, HR 1.54, 95% CI 1.04–2.28). Similarly, increasing tumour stage was associated with shorter RFS and OS (RFS log-rank test, *p* = 0.019; Cox test, *p* = 0.020, HR 1.56, 95% CI 1.07–2.26; OS log-rank test, *p* < 0.0001; Cox test *p* < 0.0001, HR 1.84, 95% CI 1.32–2.57). In addition, patient age above median at the time of diagnosis was associated with shorter RFS and OS (RFS log-rank test, *p* = 0.006; Cox test, *p* = 0.006, HR 1.67, 95% CI 1.16–2.42; OS log-rank test, *p* = 0.026; Cox test *p* = 0.027, HR 1.46, 95% CI 1.04–2.03). No significant associations were observed between TP53 or EBV status and survival.

The multivariate model for OS included the following variables: patient age at diagnosis (below versus above median, median 72.3 years), postoperative T (T1–T2 versus T3–T4), tumour stage (I–II versus III–IV) and MMR status (MSS versus MSI, for intestinal tumours only). Among intestinal tumours, MSI status was found to be predictive for longer OS (Cox test, *p* = 0.015, HR 0.46, 95% CI 0.25–0.86) while patient age above median (Cox test, *p* = 0.009, HR 1.57, 95% CI 1.12–2.21) and increasing tumour stage (Cox test, *p* = 0.036, HR 1.50, 95% CI 1.03–2.18) were predictive for shorter OS. In diffuse-type tumours, patient age above median remained as a single predictive factor for shorter OS (Cox test, *p* = 0.030, HR 2.29, 95% CI 1.08–4.83) (Table 4).

**Table 4:** Recurrence-free survival (RFS) and overall survival (OS) of patients with intestinal- or diffuse-type gastric adenocarcinoma

	Univariate survival analysis for RFS						Univariate survival analysis for OS						Multivariate survival analysis for OS			
	Number of patients	RFS, median (months)	*p* value^a^ (log-rank test)	*p* value^a^ (Cox’s test)	HR	95% CI	Number of patients	OS, median (months)	*p* value^a^ (log-rank test)	*p* value^a^ (Cox’s test)	HR	95% CI	Number of patients	*p* value^a^ (Cox’s test)	HR	95% CI
Intestinal																
Age																
Below median (ref)	67	56.6	*0.006*	*0.006*	1.67	1.16–2.42	80	37.6	*0.026*	*0.027*	1.46	1.04–2.03	78	*0.009*	1.57	1.12–2.21
Above median	92	26.8					102	27.6					100			
Patient sex																
Female	58	45.6	0.130	0.131	0.75	0.51–1.09	67	33.8	0.153	0.155	0.78	0.55–1.10				
Male (ref)	101	32.9					115	30.0								
Location																
Proximal	64	28.5	0.128	0.129	1.32	0.92–1.90	73	27.0	0.196	0.197	1.24	0.89–1.73				
Distal (ref)	95	38.2					109	32.7								
Grade																
Grade 1 (ref)	16	24.4	0.752				17	32.7	0.679							
Grade 2	78	28.5		0.696	1.13	0.62–2.07	88	27.0		0.380	1.30	0.72–2.36				
Grade 3	65	44.6		0.954	0.98	0.53–1.82	77	34.3		0.464	1.25	0.69–2.29				
T																
T1–T2 (ref)	43	67.3	*0.045*	*0.046*	1.53	1.01–2.32	47	66.8	*0.030*	*0.031*	1.54	1.04–2.28	52	0.184	1.36	0.87–2.12
T3–T4	116	28.5					135	27.6					174			
Stage																
I–II (ref)	108	38.2	*0.019*	*0.020*	1.56	1.07–2.26	114	37.3	*< 0.0001*	*< 0.0001*	1.84	1.32–2.57	110	0.036	1.50	1.03–2.18
III–IV	51	22.6					68	17.5					68			
TP53																
Aberrant	84	28.2	0.148	0.150	1.30	0.91–1.86	97	25.4	0.169	0.170	1.26	0.91–1.75				
wt (ref)	71	45.6					81	44.1								
EBV																
Positive	15	60.5	0.309	0.311	0.73	0.39–1.35	17	53.2	0.344	0.345	0.76	0.43–1.35				
Negative (ref)	140	32.6					161	28.8								
MMR																
MSI	17	124.5	0.110	0.114	0.62	0.34–1.12	17	124.6	*0.040*	*0.043*	0.54	0.30–0.98	161	*0.015*	0.46	0.25–0.86
MSS (ref)	138	32.6					161	28.7					17			
EBV neg, TP53 wt, MSS																
Yes	42	37.3	0.599	0.599	1.11	0.75–1.66	50	28.8	0.414	0.415	1.16	0.81–1.67				
No (ref)	113	32.6					128	29.0								
Diffuse																
Age																
Below median (ref)	32	22.2	0.112	0.118	1.89	0.85–4.21	38	17.4	*0.037*	*0.041*	2.06	1.03–4.14	38	*0.030*	2.29	1.08–4.83
Above median	9	15.0					12	8.74					12			
Patient sex																
Female	24	22.2	0.732	0.732	0.88	0.41–1.86	29	17.9	0.400	0.401	0.75	0.39–1.46				
Male (ref)	17	16.1					21	15.0								
T																
T1–T2 (ref)	8	NA	*0.021*	*0.031*	3.76	1.13–12.5	8	NA	*0.015*	*0.024*	3.91	1.19–12.8	8	0.118	2.77	0.77–9.95
T3–T4	33	14.9					42	15.0					42			
Stage																
I–II (ref)	24	33.3	*0.047*	0.052	2.13	0.99–4.56	26	33.6	0.066	0.070	1.85	0.95–3.59	26	0.186	1.66	0.78–3.54
III–IV	17	13.4					24	12.9					24			
TP53																
Aberrant	6	7.43	0.617	0.618	1.31	0.45–3.85	8	7.23	0.905	0.905	0.94	0.36–2.47				
wt (ref)	34	16.6					40	17.4								
EBV																
Positive	0		NA	NA			0		NA	NA						
Negative (ref)	40	16.6					48	16.0								
MMR																
MSI	0		NA	NA			0		NA	NA						
MSS (ref)	40	16.6					48	16.0								
EBV neg, TP53 wt, MSS																
Yes	34	16.6	0.617	0.618	0.76	0.26–2.23	40	17.4	0.905	0.905	1.06	0.40–2.78				
No (ref)	6	7.43					8	7.23								

*EBV*, Epstein-Barr virus; *MMR*, mismatch repair; *MSI*, microsatellite-instable; *MSS*, microsatellite-stable; *NA*, not applicable; *ref*, reference; *wt*, wild-type

^a^ *P* values < 0.05 are considered statistically significant and shown in italics

## Discussion

In this study, we describe a straightforward method for molecular classification of gastric cancer applicable for both clinical diagnostics and research purposes. With an emphasis on the intestinal-type adenocarcinomas, we have used IHC and ISH to define four subgroups of gastric adenocarcinomas with distinct molecular and clinical characteristics.

The recent molecular profiling studies have mainly been conducted in either Western [5] or Asian populations [4, 6–8]. The TCGA study contains patients from various geographical regions and among them they did not find any significant difference in the prevalence of the different molecular subtypes between the East-Asian group (Vietnam and South Korea) and the overall group [3]. Among the intestinal-type tumours, the frequency of EBV positive adenocarcinomas in our Finnish study population that represents Caucasian genetic background was 9.1%, which is in line with the results of 3.0–9.5% from other studies showing no clear difference between the geographical regions [3, 4, 6, 8]. The frequency of intestinal-type MSI tumours was 10.2% in our cohort which is similar to 9.2% found by Kim et al. (2016) but somewhat less than 24.5–26.0% found in other studies with mixed patient populations [3, 4]. The presence of aberrant TP53 expression was found in 55.4% of the intestinal-type tumours in our study. This is close to the 53.1% of the TCGA study but somewhat different from the 31.3% found by Cristescu et al. (2015) or 67.3% by Kim et al. (2016). Still, no clear difference can be seen between the different geographical regions.

The molecular analyses of gastric adenocarcinomas have shown that the intestinal- and diffuse-type tumours are distinguishable from each other also at the molecular level, and the intestinal-type tumours have more diverse molecular profiles than the diffuse-type tumours, which are the predominant subtype in the “genomically stable” category [3, 4]. Therefore, the intestinal-type adenocarcinomas have been the main focus of our analyses, and a subset of diffuse-type tumours has served as a reference group for other publications. We have shown that the intestinal-type tumours rarely contain aberrant E-cadherin expression; and that by beginning with the Laurén classification, we could concentrate 25/28 (89.3%) of the tumours with aberrant E-cadherin expression into the diffuse subgroup. Notably, none of the EBV negative, MSS and TP53 wt intestinal-type tumours showed aberrant E-cadherin expression. This suggests that some other biomarker could be more specific than E-cadherin in detecting the intestinal-type tumours not characterised by EBV positivity, MSI or TP53 aberrations. In all, these observations imply that the histological subtype can be used as a starting point for a clinically applicable method for molecular classification.

Among the diffuse-type tumours, the comparison of our results to the other studies should be considered as suggestive as we have analysed only a subset of the diffuse-type tumours diagnosed during the study period. The frequency of EBV positivity has been reported to be 3.8–9.8% among diffuse-type tumours [3, 4, 6, 8], but in our study none of the diffuse-type tumours was found to contain EBV positivity. Similarly, we did not detect MSI in the diffuse-type tumours. In other studies, the proportion of MSI diffuse-type tumours has been reported to be relatively low (3.8–8.7%) [3, 6] with the exception of 16.9% by Cristescu et al. (2015). The frequency of aberrant TP53 expression in our study was 19.2% among the diffuse-type tumours, which is quite similar to the observed frequencies of 26.1–27.5% [3, 4] but notably different from 53.8% reported by Kim et al. (2016).

The subgroup of EBV negative, MSS and wild-type TP53 tumours comprised 28.0% (52/186) of the intestinal-type tumours and 80.8% (42/52) of the diffuse-type tumours in our study population. In the TCGA study, this pattern was found in 23.0% (45/196) of the intestinal-type and 56.5% (39/69) of the diffuse-type tumours. In the ACRG study, the respective frequencies are 38.0% (57/150) for the intestinal-type tumours and 45.8% (65/142) for the diffuse-type tumours.

We could demonstrate the association of EBV positivity with male patients and the location of the tumour in the gastric corpus as previously shown by Ahn et al. (2017). Additionally, EBV positivity was found to be associated with poor histological differentiation among the intestinal-type tumours. Distally located tumours were more often characterised by MSI than proximal tumours, which confirms the results obtained by the TCGA and Cristescu et al. (2015). We could also show that *EGFR* and *HER2* gene amplifications were most common among the intestinal-type tumours with EBV negativity, MSS and TP53 aberration, of which 17.3% (17/98) were *EGFR* and 15.3% (15/98) *HER2* amplified. Notably, also among the EBV negative, MSS and TP53 wild-type tumours, the proportion of these amplifications, 15.4% (8/52) for both genes, was substantial. In the TCGA study population, 10.6% (9/85) of the EBV negative, MSS and TP53 aberrant tumours contained *EGFR* and 34.1% (29/85) contained *HER2* gene amplification. Similar to our results, among the EBV negative, MSS and TP53 wild-type tumours, the proportion of *EGFR* gene amplification was 11.1% (5/45) and *HER2* gene amplification 13.3% (6/45) [3, 16, 17]. In our study, the co-localisation of aberrant TP53 expression together with *EGFR* or *HER2* gene amplification was noticed to be more common in the proximally than distally located intestinal-type tumours, which is in line with the results from a recent characterisation of oesophageal carcinomas showing strong genomic similarities between oesophageal adenocarcinomas and CIN-type gastric adenocarcinomas according to the TCGA classification [18]. Additionally, we observed that patients with MSI tumours had longer survival than patients with MSS tumours both in the univariate and multivariate analysis, which is consistent with earlier findings [4, 7].

Our classification method concentrated on the intestinal subtype of gastric adenocarcinoma, where receptor tyrosine kinase (RTK) copy number alterations are more common than in diffuse-type tumours. In the TCGA study population, all tumours with either *EGFR* or *HER2* gene amplification were observed to have intestinal-type histology [3]. However, we also examined a subset of diffuse-type tumours in order to make comparisons with the results obtained by other studies. Most likely, due to this selection, we did not find EBV positivity or MSI among the diffuse-type tumours in our study population.

In order to increase the reliability of the TMA analysis, we have examined four tissue cores from each tumour. For the *EGFR* and *HER2* SISH, we have used whole slide sections in order to account for the potential spatial heterogeneity of the gene amplifications. Additionally, we have examined the expression of all four MSI markers instead of only one in order to increase the reliability of the classification into MSI or MSS tumours. Especially in the MSI classification, there is variation in the reported prevalence of MSI phenotype, which may partly be related to technical issues in performing and interpreting the IHC reactions. Moreover, some variability is inevitably related to using TP53 IHC as a surrogate marker for *TP53* gene mutations.

Our classification method differs from that suggested by Park et al. (2016) in that our initial division was based on the Laurén classification. After that, we continued by categorising the tumours first by EBV and second by MSI reactivity. Finally, we considered the presence of TP53 aberrations.

Our method was able to determine four distinct subgroups among the intestinal-type tumours without considerable overlapping of the molecular markers. Among the diffuse-type tumours in our material, EBV positivity, MSI and aberrant TP53 expression were mutually exclusive. Among the intestinal tumours, only one EBV positive tumour showed aberrant TP53 expression and four tumours with MSI showed aberrant TP53 expression. EBV positivity and MSI were mutually exclusive also among the intestinal-type tumours, which is in line with other studies [3, 6]. Only 3/183 intestinal-type tumours (1.6%) had aberrant E-cadherin expression, and even they could be classified as either EBV positive, MSI or TP53 aberrant. The proportion is comparable to the observed 4.1% (8/196) of the intestinal-type tumours with E-cadherin mutations in the TCGA study.

A few articles have recently been published describing different algorithms implementing the molecular classification system proposed by TCGA into clinical practice [5–8]. However, only few studies have utilised the Laurén classification and none of them have based their proposed algorithm on the histological subtype of the tumours. In one study, differentially expressed genes were compared by microarray analysis between intestinal and diffuse gastric cancers, and a 40-gene signature was created to serve as a prognostic tool [19]. As RTK amplifications are known to be more prevalent among the intestinal-type tumours, the histological subtype could be a relevant factor to take in to account when investigating new RTK-targeting therapies for gastric cancer [3]. In only one of these recent studies, the evaluation of both *EGFR* and *HER2* gene amplification status has been performed by ISH [7]. However, the SISH procedure was carried out on TMA slides, which potentially does not account for the tumour heterogeneity. So far, anti-EGFR antibodies have not shown survival benefit in phase III trials including gastric or oesophagogastric cancer patients [20, 21]. One reason for this may be that the screening methods used have not been able to identify the subgroup of patients possibly responsive to anti-EGFR treatment. In future clinical trials, the application of new classification methods combining both histological and molecular information may become necessary in order to improve the clinical benefit obtained by new targeted therapies.

In conclusion, we have demonstrated that gastric adenocarcinomas can be classified into biologically and clinically relevant subgroups by using a straightforward and clinically applicable method based on the Laurén classification together with immunohistochemistry and in situ hybridisation.
